# New Technologies and Hybrid Surgery for Atrial Fibrillation

**DOI:** 10.5041/RMMJ.10116

**Published:** 2013-07-25

**Authors:** Mark La Meir

**Affiliations:** University Hospital Brussels, Belgium; and University Hospital Maastricht, The Netherlands

**Keywords:** Stand-alone atrial fibrillation, catheter ablation, hybrid procedure

## Abstract

The Cox maze III and Cox maze IV procedures are surgical solutions for the treatment of symptomatic stand-alone atrial fibrillation. Despite their proven efficacy, these procedures have not gained widespread acceptance because of the invasiveness, complexity, and technical difficulty. Endocardial pulmonary vein isolation is the cornerstone of percutaneous catheter ablation for atrial fibrillation. It is currently accepted as an invasive therapy, if rhythm control has failed using antiarrhythmic drugs or electrical cardioversions. Pulmonary vein isolation is reported to be effective in 60%–85% of patients with paroxysmal atrial fibrillation and in 30%–50% of patients with persistent atrial fibrillation. A second or third ablation is often necessary to achieve these results, and complications may occur in up to 6% of patients.

Surgical treatment of atrial fibrillation has seen important improvements in the last decade. New technologies have simplified creation of transmural lesions on the beating heart through a less-invasive, thoracoscopic procedure. This allows for pulmonary vein isolation, isolation of the posterior wall, and left atrial appendage exclusion—usually combined with ganglionic plexi evaluation and destruction. Nonetheless, it is still uncertain whether these procedures are effective in restoring permanent sinus rhythm since transmurality of a lesion set cannot be guaranteed with current ablation catheters on the beating heart.

In an attempt to limit the shortcomings of an endo- or an epicardial technique, a hybrid approach has recently been introduced. This approach is based on a close collaboration between the surgeon and the electrophysiologist, employing a patient-tailored procedure which is adapted to the origin of the patient’s atrial fibrillation and takes into consideration triggers and substrate. Using a mono- or bilateral energy source, a thoracoscopic epicardial approach is combined with a percutaneous endocardial ablation in a single-step or in a sequential-step procedure.

This article provides our experience and an overview of the current knowledge in the hybrid treatment of stand-alone atrial fibrillation.

A combined endocardial and epicardial procedure for the treatment of atrial fibrillation is an objective and impartial way the cardiac surgeon and the electrophysiologist can explore as a team, an approach to achieve a superior long-term cure rate, achieved with a single-session procedure.

The basic concepts of cardiac surgery and electrophysiology in atrial fibrillation treatment are often obscured by different strategies that lead to conflicting trends and therefore misunderstandings. From the electrophysiologist’s viewpoint, ablation of the pulmonary veins with proof of an acute bidirectional electrical isolation is the cornerstone of most ablation strategies. On the surgical side, the foundation of a successful atrial fibrillation procedure is still a Cox maze procedure on the arrested heart, with no electrophysiological confirmation of the effect and quality of the lesion set. These distinctive characteristics of the two treatment platforms can only be changed if both the electrophysiologist and the cardiac surgeon are willing to accept their methodological limitations. If in each group we are able to confront this, then the necessity of a link between the two disciplines will become clear. In order to realize this multidisciplinary approach we must first understand the current limitations of energy delivery in the left and right atrium. The benefits of this multidisciplinary approach will enhance the controlled power delivery to targeted cardiac tissue and the accuracy of the visualization and mapping of the ablated tissue in both atria. Fundamental questions, like the necessity of a continuous and transmural lesion, will no longer be unanswered. We can map triggers and substrate at both the endocardium and epicardium, thus improving our understanding of the mechanisms of atrial fibrillation, and confirm lesion transmurality from both sides, with a single combined procedure.

Recent electrophysiology literature shows that long-lasting endocardial catheter isolation of the pulmonary veins, whether achieved with radiofrequency energy or cryo-thermia, remains challenging.^1^ Because of this limitation it is not clear whether complete circumferential antral ablation is necessary for successful pulmonary vein isolation in patients with paroxysmal atrial fibrillation, and it is accepted that non-circumferential antral ablation may achieve similar success rates with shorter procedure and ablation times than circumferential ablation. Therefore, attention could be focused on producing permanent lesions rather than on completing antral encirclement after isolation is achieved.^2^–^4^

This basic philosophy was the rationale of our initial experience with the minimally invasive surgical treatment of lone atrial fibrillation. In 2005 we developed a technique using a monolateral right thoracoscopic approach. The procedure consisted of the creation of a box lesion set to encircle all pulmonary veins with a catheter that used microwave energy to ablate left atrial tissue. At that time, this device was the only commercially available thoracoscopic minimally invasive surgical ablation tool.^5^,^6^ The concept and development of the box lesion as a minimal lesion set was based on several factors but, most importantly, a consequence of the absence of provocative electrophysiologic mapping and testing during the surgical procedure. The rationale was to maximize the number of excluded triggers by isolating the four pulmonary veins; try to obtain an important substrate modification by isolating the posterior wall of the left atrium; reduce the critical mass of the left atria; denervate the four major ganglionated plexi; and to ablate the ligament of Marshall. A major drawback for a monolateral right-sided approach was the lack of opportunity to exclude or occlude the left atrial appendage safely. Since the left atrial appendage is largely responsible for thrombo-embolic events in patients with atrial fibrillation, and can be part of the substrate responsible for atrial fibrillation, it could be preferable to occlude or exclude the left atrial appendage in a subgroup of atrial fibrillation patients. We therefore developed a technique with a monolateral left-sided approach for patients when isolation and exclusion of the left atrial appendage were deemed necessary. Freedom of atrial fibrillation at 1 year was 73% for the combined group of right- and left-sided interventions. A complementary endocardial approach was performed at 6 months in 18 patients.^7^ Since the success rate at 2-year follow-up was unsatisfactory,^8^ we changed the energy source from microwave to monopolar radiofrequency energy. Realizing that the concept of an epicardial box lesion had distinct limitations and was difficult to achieve on a beating heart (epicardial fat, heat-sink effect, power delivery of a monopolar ablation device), we combined the surgical procedure with a simultaneous endocardial electrophysiology procedure. A single-session hybrid atrial fibrillation procedure was born. For the first time, we could study the effect of an epicardial ablation on the endocardium in a human being as well as see the epicardial effects of an endocardial ablation, during the same procedure. Using this approach we could demonstrate that after epicardial creation of a box lesion with microwave or radiofrequency there was a conduction delay from the pulmonary veins and the posterior wall of the left atrium, but no exit or entrance block. This incomplete epicardial surgical ablation line necessitated a complementary endocardial isolation of one or more pulmonary veins and/or the roof and inferior line.

The importance of these findings was twofold: first, we proved that the concept of combining a percutaneous endocardial approach with a thoracoscopic epicardial approach was safe and technically feasible and, secondly, that creation of a continuous transmural box lesion from the epicardium with a monopolar energy source was not possible. Even with satisfactory clinical results, transmurality and continuity of epicardial lesions could not be assured. This could probably explain the relatively low success rate at long-term follow-up. Again we had to change our strategy. We decided to focus first on an antral epicardial isolation of the pulmonary veins. Our belief was that in order to be an alternative for an endocardial ablation, it was mandatory for the surgical portion of the hybrid approach to achieve a long-lasting antral isolation of the pulmonary veins. Since microwave and monopolar radiofrequency energy proved to be inconsistent, we had to search for an alternative. Prasad et al. showed in an animal model the potential of a bipolar radiofrequency clamp to isolate pulmonary veinselectrically.^9^ Damiano et al. studied the results of a Cox maze IV using a bipolar radiofrequency clamp and found that they were similar to the “cut and sew” Cox maze III.^10^ Thus it was assumed that a bipolar radiofrequency clamp could be able to isolate the pulmonary veins on the beating heart. However, endocardial redo procedures in patients with recurrence of atrial fibrillation who had had a thoracoscopic bipolar pulmonary vein isolation showed that in 50% there was failure to isolate one or more of the pulmonary veins.^11^ We demonstrated that mechanical clamping-induced ischemia could be responsible for these failures.^12^ Therefore, combining a bilateral thoracoscopic approach with antral isolation of the pulmonary veins, followed by an endocardial mapping and touch-up ablation, at least 30 minutes after the epicardial ablation, could avoid incomplete isolation of the pulmonary veins. Building upon this antral isolation of the pulmonary veins, we then could focus on the creation of linear lesions connecting the superior pulmonary veins and the inferior pulmonary veins using a bipolar unidirectional linear pen, thus achieving compartmentalization of the posterior left atrium.

The group of Damiano demonstrated in an animal model the potential risk of incomplete lesions using these devices.^13^ Our clinical experience confirmed their findings: in 23% of patients, the epicardial lines created with these linear ablation devices were not transmural and necessitated an endocardial touch-up ablation, demonstrating the importance of power application and mapping during the catheter treatment of atrial fibrillation.^14^ The possibility to perform such an endocardial touch-up to render epicardial lesions completely transmural is one of the major advantages of this dual epicardial–endocardial approach.

This hybrid procedure also appeared to be an advantage in performing a redo catheter ablation procedure by offering the possibility to map the patient endocardially first. An important percentage of patients that are sent for an epicardial treatment of atrial fibrillation will have had a previous endocardial procedure, mostly pulmonary vein isolation. Knowing which veins have been isolated, and which have not, can have important consequences for the treatment strategy. If all pulmonary veins have been electrically isolated, the epicardial procedure should be focused on linear lesions to compartmentalize the posterior left atrium and (mostly) exclusion of the left atrial appendage. In these cases the thoracoscopic procedure can be limited to the left-sided approach. If the pulmonary veins have been isolated on the left side, the thoracoscopic procedure could be limited to the right side.

Starting from a single-sided thoracoscopic procedure with a monopolar energy source on the beating heart, we were initially confronted with the surgical and technical limitations of the ablation devices and the procedure. A simultaneous endocardial approach seemed mandatory to understand the limitations of our minimally invasive approach. Initially setting up this collaboration with the electrophysiologist was challenging. Some of the obstacles we had to overcome were: trying to understand our common goals, organizing the availability of the different multidisciplinary teams, criteria for selection of patients, deciding where the procedure should be performed, and the sequence of the procedure. Evaluation of our findings acutely and over time has made necessary several changes to our approach and choice of ablation technologies and devices. This could only be achieved through a genuinely open-minded team approach that remained critical regarding the achieved success and also a willingness to take a retrospective view to compare this new approach to other more standard procedures.

## SINGLE-SESSION HYBRID PROCEDURE VERSUS PERCUTANEOUS CATHETER ABLATION

The reported success rate of percutaneous catheter ablation of paroxysmal atrial fibrillation with a single procedure ranges from50% to 80%.These results are even lower for patients with persistentatrial fibrillation (30% to 50%). The differences in success rates could be explained by a variety of reasons, including the experience of the center, the ablation strategy, the technology, the follow-up criteria, and other variables. A major concern is the significant recurrence rate after initial complete pulmonary vein electrical isolation, necessitating repeat interventions to achieve long-term cure of atrial fibrillation (even in high-volume centers). Recovered pulmonary vein conduction after initial acute circumferential pulmonary electrical isolation is the dominant rationale for recurrent atrial fibrillation and atrial tachyarrhythmias.^15^ The problem of durability of contiguous and transmural ablation lines in percutaneous transvenous endocardial procedures is related to multiple factors: the procedure is performed with the help of virtual imaging (fluoroscopy and three-dimensional mapping) limiting actual anatomical accuracy; the permanent tissue effects of ablation will depend on adequate and stable catheter tip to tissue contact; and the actual necessary parameters of energy delivery are difficult to define in an environment of circulating blood. These issues can be addressed by epicardial application of a bipolar radiofrequency clamping device and should therefore result in more consistent antral lesions and isolation of the pulmonary veins. With an epicardial approach, direct anatomical visualization and stable device tip to tissue contact are obtained. Furthermore, clamping the tissue between the two jaws excludes the effect of circulating blood on delivery of power, thereby eliminating the heat-sink cooling effect to the tissue. However, anatomical limitations and epicardial fat can still prevent transmurality. The epicardial approach is anatomical and fast, and creates long contiguous lines. It also partially eliminates the ganglionated plexi and allows for access to the left atrial appendage, which can be more safely excluded. This epicardial approach is therefore complementary to the endocardial procedure which can test and confirm the quality of the lesion set, can make a left and right isthmus line, and can also eliminate complex fractionated electrograms. These complementarities by themselves also have the potential to reduce complications related to both individual procedures. On the epicardial side, the combined approach avoids the necessity for a more invasive surgical procedure such as the Cox maze. The limited epicardial electrophysiologic end-points of acute exit and entrance block can be improved upon by more effective and complete endocardial mapping. On the endocardial side, the combined approach will avoid the risk for tamponade, esophageal fistula, phrenic nerve injury, and prolonged fluoroscopy. The risk of thrombo-embolic events with an epicardial approach is close to zero. By replacing most of the endocardial ablations with epicardial ablations, the total number of thrombo-embolic events will be reduced.^16^ In short, the possibility to perform endocardial mapping of the epicardial lesion set, as well as the ability to map and touch-up endocardially an incomplete lesion, is what makes the hybrid procedure successful.

## SEQUENTIAL HYBRID PROCEDURE VERSUS PERCUTANEOUS CATHETER ABLATION

Mahapatra et al. published their initial experience with surgical epicardial catheter and endocardial ablation for atrial fibrillation carried out in two sequential steps, but during the same hospitalization.^17^ Fifteen patients with persistent or long-standing persistent atrial fibrillation who failed at least one catheter ablation and one antiarrhythmic drug were treated. This group was matched categorically to 30 patients who had previously failed at least one catheter ablation and underwent a repeat catheter ablation. Five sequential hybrid patients had seven inducible atrial flutters that were mapped and ablated. After a mean follow-up of 20.7±4.5 months, 86.7% of patients of the sequential group were free of any atrial arrhythmia and off all antiarrhythmic drugs, compared to 53.3% of the catheter-alone patients. The authors concluded that, for patients with atrial fibrillation who have failed catheter ablation, sequential minimally invasive epicardial surgical ablation followed by endocardial catheter-based ablation has a higher early success rate than repeat catheter ablation alone.

This staged approach may have logistical advantages over a combined, single-session hybrid procedure. A potential drawback could be associated edema of the tissue post-ablation with difficulty to test and re-ablate the same area after a short waiting period. A second problem could be the possible increase of complication rates since the endocardial and epicardial procedures are performed separately.

## HYBRID PROCEDURE VERSUS SURGICAL ABLATION

By replacing the incisions of the traditional Cox maze III procedure with less invasive linear lesions of ablation using bipolar radiofrequency energy, Damiano et al. introduced the Coxmaze IV procedure. This procedure requires cardiopulmonary bypass and at least one small right thoracotomy. The freedom fromatrial fibrillation recurrence was 84% at 2 years for patients off antiarrhythmic drugs.^10^ These figures are comparable with our results, although, in the case of the hybrid procedure, no cardiopulmonary bypass is needed and neither is a thoracotomy. We know that none of the existing surgical ablation technologies (even bipolar radiofrequency energy) can guarantee complete transmurality.^11^ We solved this limitation by the addition of endocardial mapping and, in the case of incomplete lesions, application of radiofrequency energy endocardially. Another shortcoming of the surgical approach is the inability to locate atrial fibrillation triggers precisely, or to map atrial tachycardia and re-entrant arrhythmias known to occur during atrial fibrillation ablation procedures. Utilization of a hybrid procedure makes it is possible to perform extensive mapping in order to tailor the lesion set to the patient’s diagnostic characteristics. Finally, if the surgical procedure is performed epicardially on the beating heart, with current devices, it is technically impossible to create a linear lesion across the left and right isthmus towards the tricuspid and/or mitral valve annulus. Both of these lesions can be performed with a hybrid approach. Nonetheless only a randomized study with a significant number of patients will be able to demonstrate a preferred technique according to the classification of atrial fibrillation and its complication rate.

## HYBRID PROCEDURE VERSUS SURGICAL ABLATION WITH EPICARDIAL MAPPING

Lockwood et al. described a technique for assessing conduction block across surgical lesions based on epicardial mapping.^18^ They identified gaps in linear lesions by pacing the atrium epicardially on one side of the ablation line and mapping the direction of atrial activation on the opposite side of the lesion. Transmurality of linear lesions was also assessed by reduction of atrial electrogram potential amplitude along the linear lesion and the development of double atrial potentials along the ablation line. Using radiofrequency devices, they achieved complete block across linear lesions in the first set of radiofrequency applications in only 21%. Several factors like epicardial fat and local myocardial thickness limited the depth of penetration of radiofrequency and thus the creation of transmural lesions. After identification and localization of the gaps, epicardial ablation was repeated until complete bidirectional block across all the linear lesions was confirmed. During a hybrid procedure, provocative pacing maneuvers and mapping techniques are performed from the endocardial side. In our series, in 23% of patients we were not able to completely create a box lesion, even after identification of remaining gaps and repeating epicardial ablation. In these patients all pulmonary veins were isolated (bipolar bidirectional clamping), but the gaps were found in the connecting lesions at the roof or inferior line (bipolar unidirectional linear pen). To create contiguous transmural lesions in these areas, we had to apply endocardial unipolar radiofrequency energy. Since the connecting lesions are created with a non-clamping device, epicardial fat, tissue thickness, and the heat-sink effect are still a concern.

Krul et al. described a series of 31 patients with atrial fibrillation that were treated with thoracoscopic pulmonary vein isolation and ganglionated plexus ablation.^19^ In patients with non-paroxysmal atrial fibrillation, left atrial ablation lines were created and conduction block verified epicardially with custom-made catheters. After 1 year, they reported comparable success rates to our series (86% of patients had no recurrence and were off antiarrhythmic drugs) but had a significantly higher complication rate. Three patients had a sternotomy because of uncontrolled bleeding during thoracoscopic surgery. An important conceptual difference between both studies is that Krul et al. could only perform epicardial lesions without the possibility of add-on endocardial lesions, including endocardial touch-ups to improve transmurality, as well as performing cavo-tricuspid isthmus and left-sided mitral isthmus ablation. In addition, they could only check completeness of ablation lesions from the epicardium, which with current technology may be insufficient to show complete electrical block. In these small patient groups it is difficult to make hard conclusions when comparing two studies. However, more than half of the patients in Krul’s study had paroxysmal atrial fibrillation and all patients had 24-hour Holter monitoring after 1year. In our series most patients had persistent or long-lasting persistent atrial fibrillation and had 7-day Holter monitoring at 1 year.

## WHAT IS THE FUTURE OF HYBRID PROCEDURES FOR THE ABLATION OF ATRIAL FIBRILLATION?

Even in the best and most experienced hands, stand-alone catheter ablations for the treatment of atrial fibrillation have a significant recurrence rate, even after initial complete pulmonary vein isolation. The need for one or possibly more repeat interventions to achieve long-term cure of atrial fibrillation is not cost-effective and increases the potential complication rate to patients unnecessarily. The majority of patients prefer a single procedure if this can be achieved safely and with minimal invasiveness. There is currently no single standard approach for pulmonary vein isolation, and the electrophysiologist will have to choose among the available techniques and technologies.

The lack of end-points and current understanding of which patients benefit most by what strategy could be improved upon by a combined endocardial–epicardial procedure. In the patient population where “atrial fibrillation begets atrial fibrillation” it seems that “catheter ablation begets catheter ablation.” A single-session hybrid procedure, although initially more complex and more costly, may lead to a higher cost-efficiency and lower complication rate because of a higher cure rate. Understanding that treatment of atrial fibrillation is mandatory because of the high costs related to the prevalence and persistence of atrial fibrillation and its associated risk of stroke despite medication, invasive therapies could become a serious economic burden. Reducing the surgical invasiveness and improving the quality of the endocardial ablation lines will increase success rates, the number of patients available for interventional procedures, and the willingness of social security and national healthcare providers to accept the costs related to these invasive treatments.

Hybrid atrial fibrillation treatment will change the working relationship between electrophysiologist, cardiac surgeon, and patient and should become a treatment option for symptomatic patients with persistent or long-lasting persistent atrial fibrillation. With increased experience it could also become the treatment of choice for patients with paroxysmal atrial fibrillation, after failed catheter ablation, or patients with increased left atrial size and important substrate modification.

## CONCLUSION

The ideal approach for atrial fibrillation should be patient-tailored, employing a procedure that is adapted to the origin of the patient’s atrial fibrillation. This procedure should take into consideration triggers and substrate modification. Therefore, the current classification of atrial fibrillation in the four categories going from paroxysmal atrial fibrillation to permanent atrial fibrillation is limited when considering an ablation strategy. Defining atrial fibrillation only utilizing a time-scale is insufficient to understand the complexity of the atrial pathology responsible for the disease. Our group has demonstrated in the lab that atrial fibrillation is not a disease coming from the endocardium or epicardium, but a disease involving the three-dimensional structure of the atria. The study and treatment of the atria can only be complete if we have simultaneous access to both the endocardium and epicardium of the beating heart. This can only be achieved through a close collaboration between the surgeon and the electrophysiologist.

The potential benefits of a hybrid procedure as a single-step or sequential ablation are important. The endocardial and epicardial approach gives us a perfect platform to study the mechanisms of atrial fibrillation and thereby may improve our understanding of the peculiarities and difficulties to treat this dynamic disease. Two complementary techniques performed in conjunction could increase long-term success rates and reduce complication rates, morbidity, and mortality related to atrial fibrillation. In a single-session procedure, the reduction of complication rates is not only because of the reduction in the number of procedures as well as single anesthesia, but more importantly through the simultaneous access during the procedure. The robustness of the approach lies in its complementary nature. In our experience almost a quarter of single-step hybrid atrial fibrillation procedures needed a touch-up with an endocardial catheter ablation to finish incomplete epicardial surgical lesions. In addition, the mitral isthmus line and the cavo-tricuspid isthmus line can only be performed when combined with an endocardial approach. In redo procedures, a knowledge of the effect of the previous endocardial procedure(s) will guide the epicardial technique.

The efficacy of this procedure as well as its superiority over catheter ablation or standard surgical techniques has to be proven by large comparative studies with long-term follow-up.
